# What’s in a Japanese *kawaii* ‘cute’ name? A linguistic perspective

**DOI:** 10.3389/fpsyg.2022.1040415

**Published:** 2022-11-08

**Authors:** Gakuji Kumagai

**Affiliations:** Department of English Language and Literature, Faculty of Letters, Kansai University, Suita, Japan

**Keywords:** phonetics, sound symbolism, place of articulation, manner of articulation, frequency code, kawaii feelings

## Abstract

While the concept termed as *kawaii* is often translated into English as ‘cute’ or ‘pretty’, it has multiple connotations. It is one of the most significant topics of investigation in behavioural science and Kansei/affective engineering. This study aims to explore linguistic (phonetic and phonological) features/units associated with kawaii. Specifically, it examines, through experimental methods, what kinds of phonetic and phonological features are associated with kawaii, in terms of the following three consonantal features: place of articulation, voicing/frequency, and manner of articulation. The results showed that the features associated with kawaii are: [labial], [high frequency], and [sonorant]. The factors associated with kawaii may include the pouting gesture, babyishness, smallness, femininity, and roundness. The study findings have practical implications due to their applicability regarding the naming of anime characters and products characterised by kawaii.

## Introduction

One of the most enigmatic Japanese words is *kawaii* (hereinafter, this word is employed without italicisation). It is often translated into English as ‘cute’ or ‘pretty’ (Yomota, 2006). According to a dictionary survey (Marcus et al., 2017), the English word ‘cute’ means ‘pleasant, attractive, clever, etc.’, and the English word ‘pretty’ means ‘pleasing, attractive, etc.’ However, more than the English words ‘cute’ and ‘pretty’, Japanese kawaii has multiple connotations. According to Nittono’s (2016) study based on the largest Japanese dictionary *Nihon Kokugo Daijiten* ‘Complete Japanese-Language dictionary’ published in 2000, ‘kawai-i (adjective)’ has different meanings; for example, it means ‘miserable’, ‘attractive’, ‘lovely’, ‘innocent’, ‘small and beautiful’, and ‘trivial’ (see also Kinsella (1995) for a study on describing the meanings of kawaii). Japanese kawaii is not equivalent to English ‘cute’ or ‘pretty’.

The polysemy of Japanese kawaii seen above makes people think that it is quite difficult to define kawaii. However, it must be noted that Japanese speakers experience situations daily wherein they feel kawaii (Nittono, 2019). This intuitive feeling of kawaii has become the focus of research in a variety of academic fields such as behavioural science (including psychology) (Nittono, 2016, 2019) and Kansei/affective engineering (Ohkura, 2017, 2019), as well as cultural studies (e.g., Kinsella, 1995; Yomota, 2006; Marcus et al., 2017).

Researchers in the field of Kansei/affective engineering have examined what kinds of physical attributes—such as shapes of artificial products, tactile texture, sound and colour—induce the feeling of kawaii among Japanese people. Ohkura (2019) showed that curved shapes, soft texture, and high-frequency sounds (around 2000 Hz) were more likely to be perceived as kawaii than angular shapes, hard texture, and low-frequency sounds, respectively. Additionally, Kiyosawa (2014) showed that the colour that induces the feeling of kawaii among Japanese females is pink including baby pink and coral pink. Moreover, Ohkura (2019) analysed mimetic words that correspond to tactile texture that people identified as kawaii. The results showed that they contained /h/, /m/, /a/, /o/, and /u/. It should be noted that Ohkura (2019) did not argue that these vowels and consonants *per se* are associated with kawaii.

Findings of past Kansei/affective engineering studies seen above (e.g., Ohkura, 2019) indicate the physical attributes that can induce Japanese people to feel kawaii. However, only a few studies to date have explored kawaii from the perspective of phonetics and phonology in linguistics. This study investigates the central question of whether there are language sounds (vowels and consonants) and prosodic units (syllables/moras and accent patterns) associated with kawaii. This question concerns sound symbolism, that is, a non-arbitrary and systematic relation between language sounds and meanings (see the sound symbolism subsection for further details). A possible reason why kawaii has not been addressed in sound symbolism research to date may be that it is difficult to account for its phonetic motivation, although many cases of sound symbolism discussed in the literature are rooted in articulatory and acoustic motivations (Kawahara, 2021a). It seems to be almost impossible to define a ‘cute’ articulator and a ‘cute’ waveform.

Against this background, the current study aims to explore linguistic (phonetic and phonological) features/units associated with kawaii (dubbed a ‘kawaii linguistics’ or ‘kawaii phonetics’ project). This research project has a number of advantages. First, it is well known that sound symbolism plays an important role in marketing (Klink, 2000; Yorkston and Menon, 2004; Klink and Wu, 2014); moreover, the outcomes of the research project might prove applicable to naming anime characters and products characterised by kawaii. Second, the phonetic and phonological features/units investigated in this project not only include vowels and consonants but also prosodic units and accent patterns; therefore, we expect that theoretical linguists, more specifically, formal phonologists, will become interested in sound symbolism (see Kawahara, 2020b for a related discussion). Third, exploring kawaii from the linguistic perspective can inspire non-linguistic researchers studying ‘kawaii’ to develop it as an interdisciplinary research project.

This paper presents a case study of the abovementioned research project; it addresses what kinds of consonants are associated with kawaii in Japanese. In phonetics and phonology, consonants are often described in terms of three characteristics: place of articulation, voicing/frequency, and manner of articulation. The current study experimentally examines what kinds of features regarding the three aspects are associated with kawaii, showing that the place-of-articulation feature is [labial], the voicing/frequency feature is [high-frequency] (or [−voiced]), and the manner-of-articulation feature is [sonorant]. Motivations for these sound symbolic associations are also discussed.

## Background

### Sound symbolism

It has generally been accepted in linguistics that the relationship between sounds and meanings is in principle arbitrary (de Saussure, 1916; Hockett, 1963). However, a growing body of research has shown that sounds are associated with particular images or meanings, referred to as sound symbolism (e.g., Hinton et al., 1994/2006; Sidhu and Pexman, 2018). For example, the vowel /a/ (as opposed to the vowel /i/) is associated with a ‘large’ image, as articulating the vowel /a/ involves a wider opening than articulating the vowel /i/ (Sapir, 1929). Additionally, low-frequency sounds are associated with a ‘large’ image, while high-frequency sounds are associated with a ‘small’ image (Ohala, 1984, 1994). These sound symbolic associations are rooted in phonetic (articulatory or acoustic) motivations.

In recent years, there has been a surge of interest in sound symbolism or a related topic such as iconicity, as the number of studies regarding these topics from the viewpoints of psychology, linguistics, and cognitive science have been increasing exponentially (Nielsen and Dingemanse, 2021). A reason for this trend is that sound symbolism, or iconicity, plays a role in language acquisition (Imai et al., 2008; Nygaard et al., 2009; Asano et al., 2015; Perry et al., 2017), language evolution (Imai and Kita, 2014; Perlman and Lupyan, 2018; Ćwiek et al., 2021), and marketing (Klink, 2000; Yorkston and Menon, 2004; Klink and Wu, 2014). Specifically, sound symbolism has received significant attention from linguists. For example, a series of the research project that explores sound symbolic associations using Pokémon names (i.e., Pokémonastics), has shown that some sound symbolic effects hold across languages (Kawahara et al., 2018; Shih et al., 2019 et seq.; see Kawahara, 2021b for an overview). Moreover, aspects of sounds and meanings in sound symbolism have recently been discussed in theoretical linguistics, namely in phonology (Alderete and Kochetov, 2017; Kumagai, 2019, 2022; Jang, 2020; Shih, 2020; Kawahara, 2020a,b, 2021c).

### Previous studies and remaining issues

Several studies have addressed phonetic and phonological factors associated with kawaii in Japanese. Kumagai (2020) conducted an experiment that presented Japanese speakers with two types of nonce words—one containing labial consonants /p, b, m, ɸ, w/, the other coronal consonants /t, d, n, s, j/. The results showed that speakers perceived the nonce words with labial consonants as more kawaii. A motivation for this ‘labial = kawaii’ association, he suggests, is a pouting gesture called ‘duck face’ or ‘bird face’, which is considered sexually enticing, as a duck face is part of ‘sexualized mouths’ (Yan et al., 2022). If a pouting gesture in a duck face is associated with attractiveness and kawaii, then it can be considered that a sound using both lips (i.e., labial consonants) is associated with kawaii. Alternatively, if a pouting gesture connotes infantility (Nomura, 2010), then it can induce people to feel kawaii. This idea is interesting because it is consistent with a study that labial consonants are used frequently in baby product names (Kumagai and Kawahara, 2020); this is detailed in the following section.

Kumagai’s (2020) study was inspired by Kumagai and Kawahara’s (2020) study on Japanese baby diaper brand names. Many baby diaper brand names in Japanese markets contain labial consonants /p, m/ (e.g., *muunii*, *meriizu*, *mamiipoko*) (Kawahara, 2017). Based on this observation, they conducted a two-alternative forced-choice task and a free elicitation task, thereby showing that names with labial consonants are appropriate for baby diaper brand names. They suggested an interesting hypothesis that labial consonants are frequent in the earlier stages of language development (Jakobson, 1941/1968; MacNeilage et al., 1997), which may have yielded the sound symbolic association between labial consonants and the image of babies.

In addition to the studies on labial consonants, a number of studies have shown that singleton [p] is the consonant most likely to express kawaii in Japanese. Kumagai (2019, 2022) showed that /p/ is often used in Japanese female nicknames; for example, Haruka Shimazaki (ex-member of a Japanese idol group, AKB48), Miho Nakayama (singer), and Riho Miaki (ex-member of a Japanese idol group, Yoshimotozaka46) were called Paruru, Miporin, and Ripopo, respectively. Moreover, some Japanese females attach the word /hime/ ‘princess’ after a real name, with /p/ used instead of the initial /h/ (e.g., Ayu (real name) + hime ‘princess’ → Ayupime ‘Princess Ayu’; this nicknaming process is called *pime-yobi* ‘prince-calling’ in Japanese). All of these nicknames replace /h/ in their original name or word with /p/, which is dubbed a sound symbolic h → p alternation to express kawaii. His experiments showed that Japanese speakers judged nonce words with /p/ to more likely be kawaii names than those without it. In addition to labiality, since [p] is a voiceless (high-frequency) consonant, Kumagai (2022) suggested that the ‘/p/=kawaii’ association is motivated by the frequency code hypothesis, such that while low-frequency sounds such as voiced consonants are associated with a ‘large’ image, high-frequency sounds such as voiceless consonants are associated with a ‘small’ image. If smallness is linked to kawaii, then a high-frequency consonant is associated with kawaii.

To summarise, the previous experiments have shown that Japanese speakers found names that contain a consonant with [labial] and/or [high frequency] to be kawaii names.

### Motivation for the experiment

The previous section showed that labiality and high frequency play a role in judgment of kawaii names. However, a number of problems remain to be solved. First, Kumagai’s (2020) experiment was a two-alternative forced-choice task, which used only coronal consonants in comparison to labial consonants; it did not examine other places of articulation such as dorsal consonants /k, ɡ/. Second, although a number of studies have shown that /p/ is associated with kawaii, indicating that high frequency plays an important role in this regard, no study on kawaii has yet made a direct comparison between high-frequency consonants such as /p, t, k/ and low-frequency consonants such as /b, d, ɡ/. To argue that both features, that is, [labial] and [high frequency], are important in the judgment of kawaii names in Japanese, these two problems need to be solved.

Third, no study has yet examined what kinds of the manner-of-articulation features are associated with kawaii. Japanese has plosives, fricatives, nasals, liquid /ɾ/ (tap), and glides in its consonant inventory. The glides are excluded from the current discussion, as they are phonotactically restricted (Labrune, 2012). This study hypothesises that consonants with high sonority (i.e., nasals and liquids) are associated with kawaii names. There are a number of reasons for this prediction. First, consonants with high sonority, or sonorants, are more frequent in Japanese female names (Shinohara and Kawahara, 2013). Second, as compared to obstruents, sonorants are more likely to undergo reduplication in the creation of nicknames of Japanese idols (e.g., Aya → Ayaya; Mayu → Mayuyu; Kawahara et al., 2019). Third, if sonorants are more appropriate for rounded shapes than obstruents in Japanese, as in the maluma-takete effect (Köhler, 1929/1947) and the bouba-kiki effect (Ramachandran and Hubbard, 2001), then we hypothesise that sonorants are more likely to be associated with kawaii than obstruents, since curved (rounded) shapes are more likely to be perceived as kawaii than angular shapes (Ohkura, 2017, 2019).

Consequently, the current study conducted a kawaii rating judgment task to reveal what kinds of place-of-articulation, frequency, and manner-of-articulation features are associated with kawaii.

## Experiment

### Task and stimuli

This study aimed to explore the phonetic and phonological features of kawaii names in terms of the following three characteristics of consonants: place of articulation, voicing/frequency, and manner of articulation. As shown in Table 1, there are three sets of stimuli for each aspect. In each column, the left subcolumn shows phonological forms and the right subcolumn shows the katakana, a Japanese syllabary. Set 1 targeted three places of articulation: labial /p, b/ vs. coronal /t, d/ vs. dorsal /k, ɡ/. Set 2 targeted high-or low-frequency consonants: high-frequency /p, t, k/ vs. low-frequency /b, d, ɡ/. Set 3 targeted four manners of articulation: plosive /d/, fricative /z/, nasal /n/, and liquid /ɾ/. As there is only one (voiced, coronal) liquid /ɾ/ in Japanese, the other three conditions in Set 3 were controlled so as to contain only a voiced coronal consonant (i.e., /d, z, n/). As already noted in the previous subsection, the glides /w, j/ were not targeted in the experiment, as they are phonotactically restricted in Japanese (Labrune, 2012). Each condition had 8 4-mora items, each of which contained two target consonants located in the first and third moras. The vowel quality in each stimulus was identical.

**Table 1:** Stimuli.

Set 1 (Experiment A): Place of articulation							
Labial		Coronal		Dorsal			
/paːpio/	パーピオ	/taːtio/	ターティオ	/kaːkio/	カーキオ		
/boːpio/	ボーピオ	/doːtio/	ドーティオ	/ɡoːkio/	ゴーキオ		
/penbio/	ペンビオ	/tendio/	テンディオ	/kenɡio/	ケンギオ		
/bonpio/	ボンピオ	/dontio/	ドンティオ	/ɡonkio/	ゴンキオ		
/benbia/	ベンビア	/dendia/	デンディア	/ɡenɡia/	ゲンギア		
/banpia/	バンピア	/dantia/	ダンティア	/ɡonkia/	ゴンキア		
/paːbisu/	パービス	/taːdisu/	ターディス	/kaːɡisu/	カーギス		
/boːbisu/	ボービス	/doːdisu/	ドーディス	/ɡoːɡisu/	ゴーギス		
**Set 2 (Experiment A): High/low frequency**							
**High-frequency (i.e., Voiceless)**		**Low-frequency (i.e., Voiced)**					
/taːkio/	ターキオ	/daːɡio/	ダーギオ				
/toːpio/	トーピオ	/doːbio/	ドービオ				
/kentio/	ケンティオ	/ɡendio/	ゲンディオ				
/pontio/	ポンティオ	/bondio/	ボンディオ				
/tenpia/	テンピア	/denbia/	デンビア				
/tankia/	タンキア	/danɡia/	ダンギア				
/kaːtisu/	カーティス	/ɡaːdisu/	ガーディス				
/poːtisu/	ポーティス	/boːdisu/	ボーディス				
**Set 3 (Experiment B): Manner of articulation**							
**Plosive**		**Fricative**		**Nasal**		**Liquid**	
/daːdio/	ダーディオ	/zaːzio/	ザージオ	/naːnio/	ナーニオ	/ɾaːɾio/	ラーリオ
/doːdio/	ドーディオ	/zoːzio/	ゾージオ	/noːnio/	ノーニオ	/ɾoːɾio/	ローリオ
/dendio/	デンディオ	/zenzio/	ゼンジオ	/nennio/	ネンニオ	/ɾenɾio/	レンリオ
/dondio/	ドンディオ	/zonzio/	ゾンジオ	/nonnio/	ノンニオ	/ɾonɾio/	ロンリオ
/dendia/	デンディア	/zenzia/	ゼンジア	/nennia/	ネンニア	/ɾenɾia/	レンリア
/dondia/	ドンディア	/zonzia/	ゾンジア	/nonnia/	ノンニア	/ɾonɾia/	ロンリア
/daːdisu/	ダーディス	/zaːzisu/	ザージス	/naːnisu/	ナーニス	/ɾaːɾisu/	ラーリス
/doːdisu/	ドーディス	/zoːzisu/	ゾージス	/noːnisu/	ノーニス	/ɾoːɾisu/	ローリス

The experiment did not use all combinations of the consonants and vowels in Japanese. For example, it did not use the combination of a consonant and the vowel /i/ or /u/ in the first mora (e.g., /piːpio/, /puːpio/). As the vowel quality was controlled across each stimulus, if [pi] or [pu] had been used in the labial condition, [ti] or [tu] must have also been used in the coronal condition. Although the sequences of [ti] and [tu] are observed only in loanwords in Japanese, words that begin with [ti] or [tu] are much less frequent than those that begin with [pi] or [pu]. Considering that words that begin with [ti] or [tu] do not sound familiar to Japanese speakers, the experiment avoided using the vowel /i/ or /u/ in the word-initial position. Instead, [ti] was used in the word-medial position.

### Procedure

This study employed an online questionnaire platform, SurveyMonkey, to collect data. Participants were recruited by using ‘the buy response function’ of SurveyMonkey. The participants were asked to sign a consent form if they assented to participate in the study; subsequently, they were asked whether they were native Japanese speakers and if they were familiar with the term ‘sound symbolism’. All of the participants stated that they were native Japanese speakers; none of them reported that they were familiar with the term ‘sound symbolism’.

The current study used orthographic stimuli by means of Japanese katakana characters, which are often used to represent loanwords in the Japanese orthographic convention. The participants were instructed that they would be presented with a name and were asked to rate it on a 4-point scale (4: *Kono namae wa totemo kawaii* ‘This name is very cute’; 3: *Kono namae wa kawaii* ‘This name is cute’; 2: *Kono namae wa amari kawaikunai* ‘This name is not very cute’; 1: *Kono namae wa kawaikunai* ‘This name is not cute’). The participants practiced rating the cuteness of a name before formally starting responding to the questionnaire.

Due to a functional limitation of SurveyMonkey, the current study was divided into two parts (Exp A and Exp B). Exp A included 40 nonce words in Sets 1 and 2, and Exp B included 32 nonce words in Set 3. Different speakers participated in each experiment (details will be presented in the next subsection).

The orders of names in each experiment were randomised for each participant. Names in the stimuli were presented at the same page, so the participants needed to scroll the page to answer all the questions. After completing all the questions, the participants were asked to provide information about their age and gender.

### Participants

A total of 150 speakers participated in Exp A, and another 150 participated in Exp B. Their age and gender are summarised in Table 2.

**Table 2:** The distribution of participants by age and gender.

	*N*	Female	Male	20–29 years old	30–44 years old	45–60 years old
Exp A	150	85	65	21	59	70
Exp B	150	65	85	16	53	81

### Statistical analysis

The results fit a Bayesian mixed-effects linear model (see Kruschke and Liddell, 2018; Franke and Roettger, 2019 for advantages of Bayesian analysis), using the package *brms* (Bürkner, 2017) in R Core Team (2022). Regarding the application of this model to experimental data, see Kawahara (2020a, 2021c) for example.

The dependent variable was score (1 to 4). The independent variable was place of articulation (labial vs. coronal vs. dorsal), frequency (high vs. low), and manner of articulation (plosive vs. fricative vs. nasal vs. liquid). The model included by-participant varying slopes, in addition to by-stimuli varying intercepts and by-participant varying intercepts.

The model was fitted using four chains each with 4,000 iterations with 1,000 warmups, thereby yielding 4,000 posterior samples. The model showed convergence, as all the R-hat values were 1.00 (i.e., less than 1.1). The results subsection below reports the coefficient estimate (β), standard error, and upper and lower 95% Credible Intervals (CI). In Bayesian analysis, if the CI includes zero, the effect is not meaningful.

## Results

The results are presented with box plots. Black diamonds represent the average score in each condition. The white boxes represent the interquartile range, thin vertical lines represent the rest of the distribution, black dots represent outliers and black horizontal lines represent the median in each condition. Table 3 summarises the results of modelling.

**Table 3:** Model summary.

		β	Est. Error	95% CI
Set 1	Intercept	0.39	0.04	[0.31, 0.47]
	dorsal (vs. coronal)	−0.09	0.05	[−0.18, 0.01]
	labial (vs. coronal)	0.10	0.05	[0.01, 0.19]
Set 2	Intercept	0.57	0.04	[0.50, 0.64]
	low-frequency (vs. high-frequency)	−0.25	0.04	[−0.33, −0.17]
Set 3	Intercept	0.32	0.04	[0.25, 0.39]
	liquid (vs. fricative)	0.30	0.04	[0.23, 0.38]
	nasal (vs. fricative)	0.16	0.04	[0.09, 0.24]
	plosive (vs. fricative)	0.02	0.04	[−0.06, 0.09]

**Figure 1:**
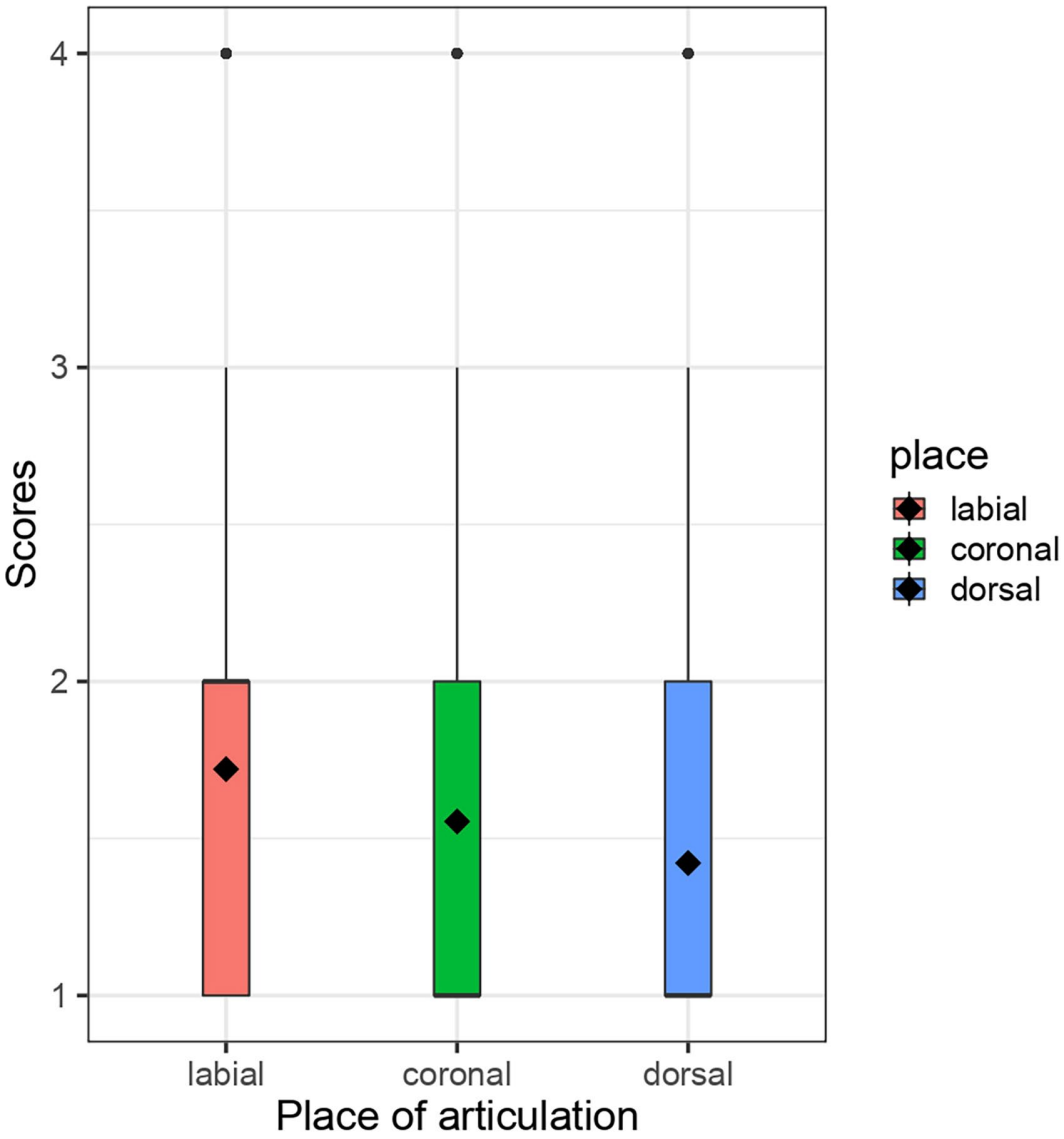
Box plots for Set 1.

Figure 1 represents the box plots for Set 1. The average score was 1.721 in the labial condition, 1.554 in the coronal condition, and 1.422 in the dorsal condition. The uppermost section in Table 3 summarises the results of modelling of Set 1. The baseline was the coronal condition, and we compared the dorsal vs. coronal conditions and the labial vs. coronal conditions. First, the difference between dorsal and coronal consonants was not credible, as the 95% CI of this coefficient estimate included zero [−0.18, 0.01]. Second, the labial vs. coronal comparison showed that the coefficient estimate was positive (β = 0.1) and its 95% CI did not include zero [0.01, 0.19], suggesting that nonce words with labial consonants were judged to be more kawaii names than those with coronal consonants.

**Figure 2:**
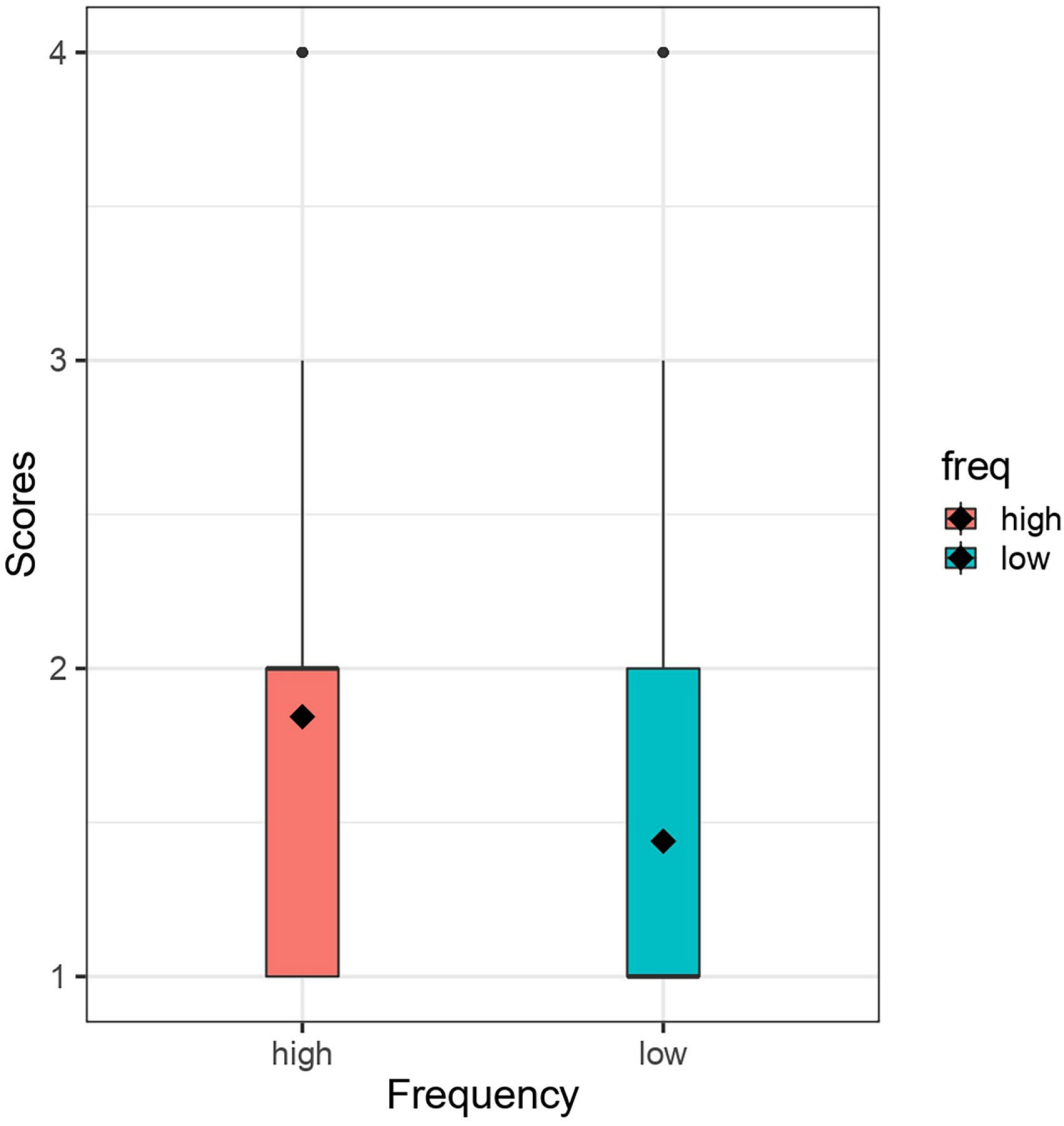
Box plots for Set 2.

Figure 2 represents the box plots for Set 2. The average score was 1.843 in the high-frequency condition and 1.439 in the low-frequency condition. The middle section in Table 3 summarises the results of modelling of Set 2. The baseline was the high-frequency consonant condition. Comparison of the two conditions showed that the coefficient estimate was negative (β = −0.25) and its 95% CI did not include zero [−0.33, −0.17], suggesting that nonce words with low-frequency consonants were judged to be less kawaii names than those with high-frequency consonants.

**Figure 3:**
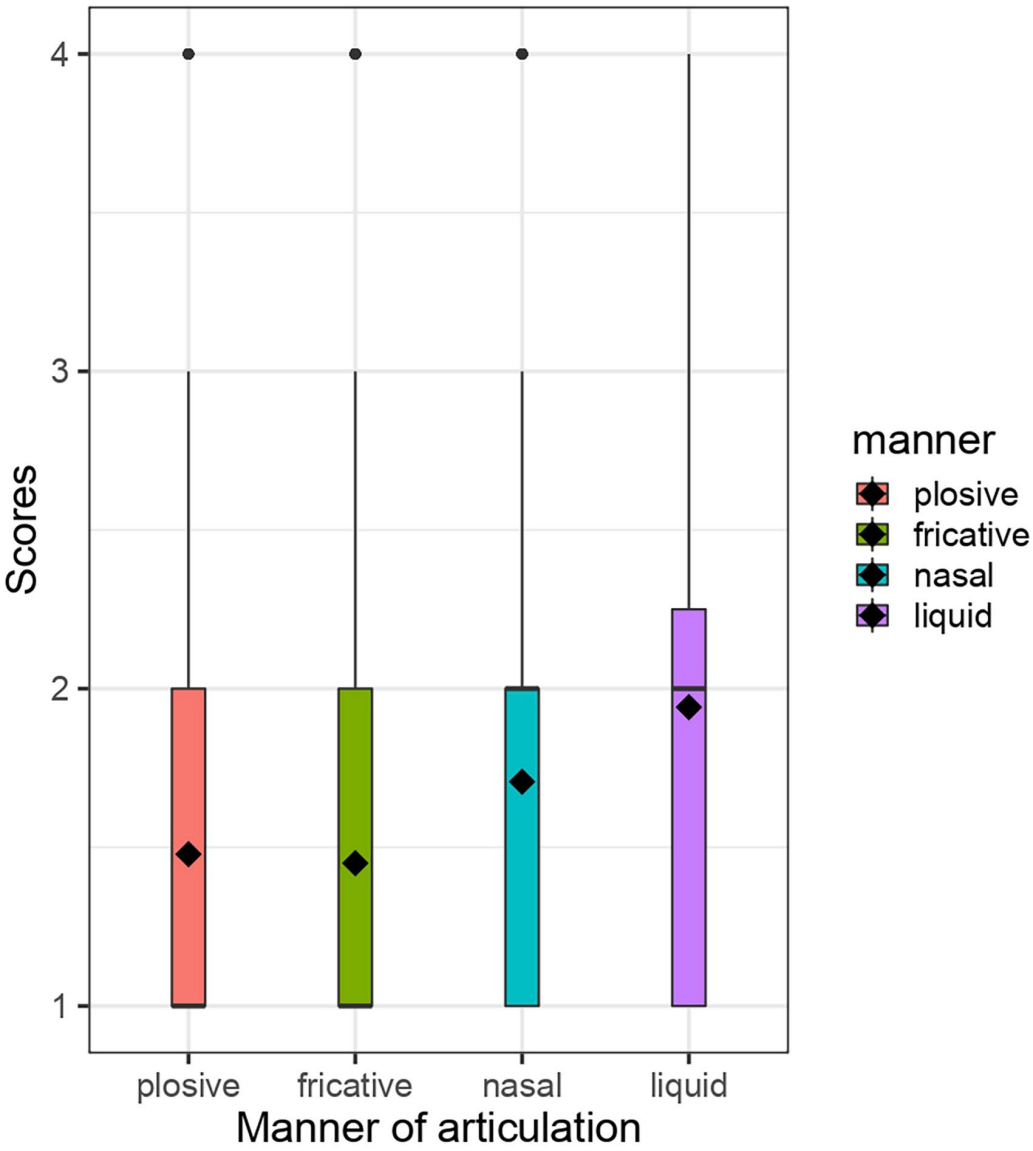
Box plots for Set 3.

Figure 3 represents the box plots for Set 3. The average score was 1.478 in the plosive condition, 1.45 in the fricative condition, 1.707 in the nasal condition, and 1.942 in the liquid condition. The lowest section in Table 3 summarises the results of modelling of Set 3. The baseline was the fricative condition, and we compared the plosive vs. fricative conditions, the nasal vs. fricative conditions, and the liquid vs. fricative conditions. First, the difference between the plosive and fricative conditions was not credible, as the 95% CI of this coefficient estimate included zero [−0.06, 0.09]. Second, the nasal vs. fricative and liquid vs. fricative comparisons showed that both of the coefficient estimates were positive (β = 0.16) (nasal) and (β = 0.3) (liquid) and both of the 95% CIs did not include zero ([0.09, 0.24] (nasal) and [0.23, 0.38] (liquid)), thereby suggesting that nonce words with nasals and liquids were judged to be more kawaii names than those with fricatives.

## Discussion

The current study showed that (1) labial consonants are more likely to be associated with kawaii than coronal and dorsal consonants, (2) high-frequency consonants are more likely to be associated with kawaii than low-frequency consonants, and (3) liquid /ɾ/ and nasal /n/ are more likely to be associated with kawaii than fricative /z/ (and plosive /d/). These results suggest that the place-of-articulation feature associated with kawaii is [labial], and the frequency feature associated with kawaii is [high frequency]. The manner-of-articulation feature requires further discussion. Since the consonant showing the highest average score was liquid /ɾ/, we can presume that the manner-of-articulation feature associated with kawaii is [liquid]. However, as the Bayesian analysis showed, nasal /n/ is more likely to be associated with kawaii than fricative /z/. Therefore, we can conclude that liquids and nasals, both of which are [sonorant], are associated with kawaii.

## General discussion

This study showed that the features of consonants associated with kawaii in Japanese are [labial], [high frequency], and [sonorant]. The motivations for the three features are briefly discussed below. The feature [labial] may be linked to a pouting gesture, that is, a gesture made using both lips can induce Japanese people to feel kawaii (Kumagai, 2020). The feature [labial] may also be linked to the image of babies, in that bilabial consonants are more frequent in the earlier phases of language acquisition (Kumagai and Kawahara, 2020). Thus, it can be said that consonants with feature [labial] can evoke the image of babies, at least in Japanese. The feature [high frequency] may stem from smallness, as the frequency code hypothesis states that high-frequency sounds are associated with smallness (Ohala, 1984, 1994). The feature [sonorant] may be connected to a number of observations on sound symbolic effects in names and shapes. Sonorants are better suited for female names or rounded shapes (Shinohara and Kawahara, 2013; Asano et al., 2015). To summarise, the factors associated with kawaii may include pouting gesture, babyishness, smallness, femininity, and roundness. It is interesting that some of these factors overlap with the factors noted by Kinsella (1995) for cute characters. She noted that ‘The essential anatomy of a cute cartoon character consists in its being small, soft, infantile, mammalian, round, without bodily appendages (e.g., arms), without bodily orifices (e.g., mouths), non-sexual, mute, insecure, helpless or bewildered’. (p. 226; *emphasis mine*). Taking the fact into consideration that Kinsella (1995) was published more than 25 years ago, it is inferred that something that evokes kawaii in the minds of Japanese speakers has not changed for at least 25 years.

As noted in the introduction section, it is well known that sound symbolism plays an important role in marketing (Klink, 2000; Yorkston and Menon, 2004; Klink and Wu, 2014). The exploration of what consonants are better suited for kawaii names is an interesting topic. Based on the above discussion, it is inferred that the consonants that induce the feeling of kawaii among Japanese people include /p/, /ɾ/, and /m/, as the first consonant /p/ is specified with [labial] and [high frequency], the second consonant /ɾ/ with [sonorant], and the third consonant /m/ with [labial] and [sonorant]. Based on his kawaii judgment experiment with Japanese speakers, Kumagai (2019) discusses whether /m/, in addition to /p/, is another consonant expressive of kawaii in Japanese, since his study results demonstrated that fewest differences existed regarding average scores between nonce words with /p/ and those with /m/. In Japanese words or character names that seem to be associated with kawaii, we find examples that contain /p/, /ɾ/, or /m/. For example, a mimetic word, or onomatopoeia, *purupuru*, is used to express something soft or something that trembles like jelly. We also find a cute character name *pomupomu purin* ‘Pom Pom Purin’, created by Sanrio. Moreover, Kawahara (2019) reported that bilabial consonants and /ɾ/ are often used in girls’ names in a popular Japanese anime PreCure, broadcast since 2004. It is expected that these consonants will prove applicable in naming anime characters or products that are characterised by kawaii.

## Concluding remarks

The current study addressed what kinds of consonant features are associated with kawaii in Japanese, from the perspective of linguistics. It concluded that the phonetic and phonological features of consonants associated with kawaii were [labial] in the place-of-articulation feature, [high frequency] in the voicing/frequency feature, and [sonorant] in the manner-of-articulation feature. The study findings have practical implications due to their applicability regarding the naming of anime characters and products characterised by kawaii.

There are a number of limitations of the current study. First, the experiment used written stimuli, rather than auditory stimuli. Previous studies of sound symbolism have shown that shapes of letters in the written stimuli can influence speakers’ judgments (e.g., Cuskley et al., 2017). Thus, it is necessary to investigate whether the experiment results hold even with auditory stimuli. Second, all of the participants in the experiment were adult Japanese speakers, so it is still unclear whether Japanese-speaking children have already acquired the sound symbolic associations in question. This study can provide a clue to resolving the question of when (and how) the sound symbolic associations emerge in the minds of Japanese speakers. Third, the experiment results did not tell us whether the phonetic and phonological features associated with kawaii show cumulative effects; namely, two instances of the same consonant in question can exhibit a stronger image than one instance. Recent research has shown that particular sound symbolic effects are cumulative (e.g., Thompson and Estes, 2011; Kawahara, 2020a,b, 2021c; Kumagai, 2022). This problem should be addressed in future research.

As already mentioned in the introduction section, Japanese kawaii is an enigmatic word in that it has multiple connotations and it is not just equivalent to the English words ‘cute’ or ‘pretty’. This aspect of kawaii has aroused researchers’ interest over the last few decades. Along this trend, the current study has revealed what phonetic and phonological features are associated with kawaii, from the perspective of linguistics. The outcome of the current study has added to a piece of our understanding with respect to Japanese kawaii.

## Data availability statement

The data on the experiment and analysis are found at the https://osf.io/anx3w/.

## Ethics statement

The studies involving human participants were reviewed and approved by Yutaka Maeda, the Ethical Review Board at Kansai University. The patients/participants provided their written informed consent to participate in this study.

## Author contributions

The author confirms being the sole contributor of this work and has approved it for publication.

## Funding

The current study was supported by JSPS Grant-in-Aid for Young Scientists (no. 19 K13164).

## Conflict of interest

The author declares that the research was conducted in the absence of any commercial or financial relationships that could be construed as a potential conflict of interest.

## Publisher’s note

All claims expressed in this article are solely those of the authors and do not necessarily represent those of their affiliated organizations, or those of the publisher, the editors and the reviewers. Any product that may be evaluated in this article, or claim that may be made by its manufacturer, is not guaranteed or endorsed by the publisher.
